# Recent advances in the understanding of renal inflammation and fibrosis in lupus nephritis

**DOI:** 10.12688/f1000research.10445.1

**Published:** 2017-06-13

**Authors:** Susan Yung, Desmond YH Yap, Tak Mao Chan

**Affiliations:** 1 Department of Medicine, University of Hong Kong, Hong Kong, Hong Kong

**Keywords:** Lupus nephritis, AKI, acute kidney injury, renal failure, SLE

## Abstract

Lupus nephritis is a potentially reversible cause of severe acute kidney injury and is an important cause of end-stage renal failure in Asians and patients of African or Hispanic descent. It is characterized by aberrant exaggerated innate and adaptive immune responses, autoantibody production and their deposition in the kidney parenchyma, triggering complement activation, activation and proliferation of resident renal cells, and expression of pro-inflammatory and chemotactic molecules leading to the influx of inflammatory cells, all of which culminate in destruction of normal nephrons and their replacement by fibrous tissue. Anti-double-stranded DNA (anti-dsDNA) antibody level correlates with disease activity in most patients. There is evidence that apart from mediating pathogenic processes through the formation of immune complexes, pathogenic anti-dsDNA antibodies can bind to resident renal cells and induce downstream pro-apoptotic, pro-inflammatory, or pro-fibrotic processes or a combination of these. Recent data also highlight the critical role of macrophages in acute and chronic kidney injury. Though clinically effective, current treatments for lupus nephritis encompass non-specific immunosuppression and the anti-inflammatory action of high-dose corticosteroids. The clinical and histological impact of novel biologics targeting pro-inflammatory molecules remains to be investigated. Insight into the underlying mechanisms that induce inflammatory and fibrotic processes in the kidney of lupus nephritis could present opportunities for more specific novel treatment options to improve clinical outcomes while minimizing off-target untoward effects. This review discusses recent advances in the understanding of pathogenic mechanisms leading to inflammation and fibrosis of the kidney in lupus nephritis in the context of established standard-of-care and emerging therapies.

## Introduction

Lupus nephritis (LN) is a severe manifestation of systemic lupus erythematosus (SLE) and is associated with a sixfold increase in mortality compared with the general population ^1^. Up to 60% of patients with SLE will develop LN at some stage during the course of disease, the percentage depending on race, ethnicity, and health-care availability ^1– 3^. LN is characterized by loss of self-tolerance, production of autoantibodies to nuclear autoantigens, immune complex deposition, and immune-mediated injury to the kidney parenchyma. Clinically, LN is characterized by periods of remission interspersed with episodes of disease activity or flares. If these inflammatory processes are not effectively and rapidly controlled, glomerulosclerosis, interstitial fibrosis, tubular atrophy, and progressive kidney failure will follow, leading to end-stage renal failure requiring renal replacement therapy. Treatment of LN has evolved considerably over the past few decades from reliance on corticosteroids alone to different combination immunosuppressive regimens, and the optimal choice is informed by the type and severity of nephritis, the phase in the natural history of disease, and patient characteristics ^4^. It has been reported that approximately 30% of patients eventually will progress to end-stage renal failure ^3, 5^. Identifying key elements in the pathogenesis of inflammation and fibrosis or important checkpoints leading to kidney damage could present opportunities for novel means of therapeutic intervention to further improve clinical outcomes.

## Overview of lupus nephritis pathogenesis

LN is initiated by the deposition of anti-double-stranded DNA (anti-dsDNA) antibody-containing immune complexes in the kidney parenchyma resulting in complement activation, infiltration of immune cells, increased activation and proliferation of resident renal cells, and immune-mediated kidney injury. The precise location of immune complexes in the glomerulus and tubulo-interstitium, their size, ability to activate complement, and the effectiveness of complex-clearing mechanisms dictate the severity of proliferative and inflammatory responses in the kidney. The glomerulus is the predominant site for immune complex deposition. Deposition within the mesangium is accompanied by mesangial hypercellularity and increased matrix synthesis, whereas their deposition in the subendothelium induces endothelial cell injury, endocapillary proliferation and infiltration of circulating myeloid cells into the kidney ^6^. Injury to endothelial cells induces the release of apoptotic microparticles that drive dendritic cell activation and prime neutrophils for NETosis, which further exacerbates inflammatory processes ^7^. Subepithelial deposits promote podocyte injury with restricted immune cell infiltration unless the glomerular basement membrane (GBM) ruptures ^8^. The most severe forms of LN (classes III and IV) are characterized by inflammatory and proliferative glomerular lesions resulting in fibrosis and loss of renal function ^9^. Immune deposits have also been detected in the tubular basement membrane in up to 70% of patients with LN, especially those with class III or IV LN, and the quantity of immune complex deposition correlates with the severity of tubulo-interstitial inflammation ^10, 11^.

Anti-dsDNA antibody production is a cardinal feature of LN, and serum levels often correlate with disease activity ^12, 13^. There is evidence that in addition to contributing to immune complex formation, pathogenic anti-dsDNA antibodies can bind to resident renal cells and induce downstream apoptotic, inflammatory, and fibrotic processes ^10, 12, 14– 20^. There is increasing evidence to suggest that the pathogenic potential of anti-dsDNA antibodies is not dependent on its interaction with dsDNA per se since dsDNA is poorly immunogenic ^21^ but is instead reliant on its poly-reactive nature and ability to bind to cross-reactive antigens on the surface of resident renal cells or constituents of their extracellular matrix ^22– 24^. Many of the studies relating to anti-dsDNA antibodies binding to resident renal cells have focused on mesangial cells and currently there are limited data on the interaction of anti-dsDNA antibodies with glomerular endothelial cells, podocytes, and proximal tubular epithelial cells (PTECs). Anti-dsDNA antibodies have also been reported to bind to nucleosomes that are released into the circulation from either circulating or intra-glomerular apoptotic cells, which are then entrapped within the GBM. Recently, Olin *et al*. demonstrated that laminin β1, an extracellular matrix component induced in resident renal cells by transforming growth factor-beta 1 (TGF-β1) during tissue fibrosis, entrapped nucleosomes in electron-dense deposits in the GBM which could serve as antigens for anti-dsDNA antibodies ^25^. Although there has been much debate on the mechanisms that mediate anti-dsDNA antibody binding to the kidney parenchyma, distinct mechanisms may operate in different renal compartments and possibly at different phases of disease.

The pathogenic mechanisms of anti-dsDNA antibodies remain controversial. Non-dsDNA-binding antibodies may also contribute to kidney injury. Dissociation between anti-dsDNA antibody deposition and LN has also been reported in experimental models of LN. In NZM2328.Lc4 mice, intra-glomerular immune complex deposition and fatal LN occurred in the absence of anti-dsDNA antibodies ^26^. Furthermore, lupus-prone mice deficient in Stat4, a transcription factor that drives Th1 responses, displayed accelerated nephritis compared with their wild-type littermates in the absence of anti-dsDNA antibodies ^27^. The mode of kidney inflammation could be more important than anti-dsDNA antibodies per se in the initiation and progression of LN ^27^. Biopsy-proven LN can occur in patients without anti-dsDNA antibodies in the circulation ^28^. Discordance between circulating anti-dsDNA antibody titres and activity of LN may also be attributed to the different dsDNA substrates used in immunoassays ^29^.

## Recent knowledge on renal inflammation in lupus nephritis

### (a) Role of resident kidney cells

The mesangium is the site for anti-dsDNA antibody-containing immune complex deposition in the glomerulus in less severe forms of LN, and mesangial immune deposits are always present in severe nephritis. We ^22^ and others ^23, 30^ have reported that anti-dsDNA antibodies can bind directly to mesangial cells through cell surface annexin II or α-actinin. The functional consequence of anti-dsDNA antibody binding to α-actinin on mesangial cells has not been defined. Binding of anti-dsDNA antibodies to mesangial annexin II resulted in the activation of mitogen-activated protein kinase (MAPK) and AKT/phosphoinositide 3-kinase (AKT/PI3K) signaling pathways and increased IL-6 secretion and annexin II expression. That anti-dsDNA antibodies can augment increased annexin II expression in mesangial cells suggests potential amplification of immune-mediated inflammation in the glomerulus ^22^. At the ultrastructural level, annexin II localized to the surface of mesangial cells and in the mesangial matrix and also with electron-dense immune deposits along the GBM ^31^. Annexin II can co-localize with immunoglobulin G (IgG) and complement 3 (C3) deposition in human and murine LN, thus underscoring the pathogenic role of annexin II and its interaction with anti-dsDNA antibodies in LN ^22^. Anti-dsDNA antibodies can induce gene or protein expression (or both) of pro-inflammatory mediators such as interleukin-1 beta (IL-1β), tumor necrosis factor-alpha (TNF-α), hyaluronan, lipocalin-2, monocyte chemoattractant protein-1 (MCP-1), (C-X-C motif) ligand 1 (CXCL1)/KC, CX3CL, and inducible nitric oxide synthase in cultured human or murine mesangial cells ^15, 17, 22, 32^. The induction of these pro-inflammatory mediators is facilitated through the binding of high-mobility group box 1 protein, engagement of Toll-like receptor 2 (TLR-2) and receptor for advanced glycation end-products (RAGE), activation of the MAPK, protein kinase C (PKC), inhibitor of kappa-light chain-enhancer of activated B cells (IκB) and nuclear factor kappa B (NF-κB) signaling pathways, and endoplasmic reticulum stress ^20, 32– 35^. Correlation between serum levels of IL-1β, IL-6, TNF-α, hyaluronan, and lipocalin and disease activity in patients with SLE further highlights the importance of these inflammatory markers in the pathogenesis of LN ^17, 36– 39^.

The severity of tubulo-interstitial inflammation and injury strongly correlates with poor renal prognosis ^40^. We reported that anti-dsDNA antibodies isolated from patients with LN during nephritic flare can induce secretion of pro-inflammatory mediators such as IL-6, IL-8, TNF-α, and MCP-1 through distinct MAPK pathways in cultured PTECs ^14, 39^ and contribute to the establishment of chemotactic gradients that permit infiltration of immune cells into the tubulo-interstitium. Bi-directional communication occurs between mesangial cells and PTECs, and inflammatory responses occurring in either kidney compartment induced by anti-dsDNA antibodies can provoke a response in the other compartment ^10^. Anti-dsDNA antibodies isolated from LN patients in remission can also induce IL-6 secretion in PTECs ^10^, a pro-inflammatory cytokine that promotes B-cell differentiation and autoantibody production. Increased IL-6 expression is observed in the kidneys of patients and mice with LN, and infiltrating monocytes/macrophages, mesangial cells, and PTECs are thought to be the predominant source. The findings suggest the possibility of subclinical inflammation, which has important implications on the choice and dose of maintenance immunosuppressive therapy. Current methods for monitoring disease activity do not reliably assess kidney inflammation and fibrosis, and the search for biomarkers which could serve such purposes is ongoing ^41, 42^.

Of the multitude of pro-inflammatory mediators synthesized by immune and resident renal cells in LN, interferon (IFN) has been implicated in both systemic and end-organ inflammation ^43^. Patients with lupus exhibit increased expression of type I IFN response genes ^43^. The importance of type I IFN in the development of LN stems from experimental and clinical studies that show reduced autoimmunity in type I IFN receptor-deficient lupus-prone mice ^44^, exacerbation of disease following administration of adenovirus encoding IFN-α to lupus-prone mice ^9, 45^, and the production of lupus-related antibodies in a significant percentage of patients whose hepatitis C was treated with IFN-α ^46^. Whether plasmacytoid dendritic cells are the main source of type I IFN remains controversial. In a murine model of anti-GBM nephritis, resident renal cells rather than infiltrating leukocytes were shown to be the dominant source of type I IFN in the kidney which augmented immune-mediated injury ^47^, possibly through TLR-3 activation ^48^. Although the investigators did not identify which cells synthesized type I IFN, it is possible that mesangial cells contribute ^49^. Regulation of type I IFN in mesangial cells is mediated at least in part by microRNA (miR)-130b, and miR-130b expression is decreased in renal specimens from patients and mice with LN ^50^.

### (b) Role of infiltrating cells

Both innate and adaptive immune systems play critical roles in systemic and intra-renal inflammatory response in LN. Intra-renal deposition of IgG-containing immune complexes alone is insufficient to initiate pathogenesis and must be accompanied by secretion of pro-inflammatory mediators and recruitment of immune cells. Multiple effector mechanisms have been identified in lupus-prone mice and these include but are not limited to Fc receptors (FcRs), type I IFN receptors, IL-6, and MCP-1 ^51– 54^. Engagement of IgG-containing immune complexes with FcRs is a critical step in the development of LN. Although FcRs are present on mesangial cells, FcR activation on circulating hematopoietic cells rather than resident renal cells initiates pathogenesis in lupus-prone mice ^51^. Also, the accumulation of apoptotic debris in hematopoietic cells has been reported to promote FcγRI-mediated PI3K signal transduction and disease development in lupus-prone mice, whereas mice deficient in FcγRI were protected ^55^. Monocytes isolated from patients with LN express increased FcγRI and exhibit increased MCP-1 secretion and chemotactic potential compared with monocytes from healthy subjects ^56^.

Increased serum type I IFN level and induction of IFN-induced gene transcript and protein signature have been observed in peripheral blood mononuclear cells (PBMCs) and renal tissue in patients with LN ^43^. Serum IFN-α in pediatric patients with lupus can induce monocyte maturation into highly active antigen-presenting dendritic cells ^57^. These myeloid cells activate naïve T cells and stimulate B-cell expansion and differentiation through B cell-activating factor (BAFF) and exacerbate autoantibody production and autoimmunity. Exogenous viruses have been suggested as a possible trigger of SLE since double-stranded RNA (dsRNA) viruses can induce IFN-α secretion in dendritic cells, but with the possible exception of Epstein-Barr virus, there are limited data to implicate viruses in SLE. Rather, self nucleic acid-containing immune complexes can activate TLRs, which mediate downstream induction of type I IFN synthesis in PBMCs. Expression of long interspersed nuclear element 1, a virus-derived nucleic acid present as a transposable element in the human genome, has recently been reported to induce type I IFN in PBMCs and may contribute to the initiation and amplification of SLE ^58^. Oxidative stress, mitochondrial dysfunction, and opsonization of apoptotic cells by complement and IgM have also been implicated in increased type I IFN production and NF-κB activation in peripheral blood lymphocytes in patients with SLE ^59, 60^. Assessment of IFN-inducible gene expression signature rather than IFN secretion may be a more sensitive method to determine IFN activation in patients with lupus and this is due to blocking antibodies that may be present in serum ^43^. The roles of IL-6 and MCP-1 in the pathogenesis of LN are well established and have been reviewed elsewhere ^61, 62^.

Chemokine production by infiltrating and resident renal cells mediates the recruitment of monocytes from the circulation and their differentiation into macrophages as they migrate to the site of injury. Macrophages can be divided into two subsets, namely M1 and M2 macrophages ^63^. M1 macrophages are classic phagocytic, inflammatory macrophages and are activated in response to IFN-γ or TNF-α to secrete large quantities of pro-inflammatory cytokines that include IL-1, IL-6, IL-12, and IL-23. M1 macrophages recruit neutrophils to the site of injury and also induce Th1 and Th17 differentiation ^64^. M2 macrophages are activated by IL-4 and can be subdivided into three groups comprising M2a macrophages that contribute to the reparative process and secretion of anti-inflammatory cytokines, M2b macrophages that are induced by immune complexes or TLR ligation, and M2c macrophages that possess anti-inflammatory and pro-fibrotic properties and play a key role in the elimination of apoptotic cells ^63, 65^. Owing to their plasticity and presence of pro- or anti-inflammatory cytokine levels within their microenvironment, macrophages can switch from an M1 to an M2 phenotype ^65^. Macrophages are key cellular determinants in the pathogenesis of LN and are an important source of key pro-inflammatory cytokines that drive autoimmunity ^66^. Their detection in renal biopsies from patients with LN is associated with crescent formation and poor renal prognosis ^67, 68^. It has been reported that M1 macrophages were more abundant in class IV LN compared with class II and V LN and were detected predominately in the glomerulus ^68^. Macrophages present in tubulo-interstitium belonged to the M2c subgroup, and their number correlated with tubulo-interstitial injury score, anti-dsDNA antibody level, and severity of renal impairment, suggesting a putative role of these cells in tubulo-interstitial injury or fibrosis ^68^. The clinical relevance of M2c macrophages is also substantiated by elevated plasma levels of sCD163 (released from M2c macrophages) during active SLE ^69^. Depletion of macrophages using a specific inhibitor to the colony-stimulating factor-1 (CSF-1) receptor in an inducible murine model of LN protected mice from renal inflammation and development of nephritis ^70^. Lupus-prone mice deficient in MCP-1 exhibited reduced numbers of interstitial macrophages, decreased proteinuria, and improvement in renal histology ^54^. The role of macrophages in experimental models of LN is dependent on the mouse model used and disease status. In the human setting, CD169 ^+^ inflammatory macrophages are present in the glomeruli of patients with LN, but not healthy subjects, and their number correlated with the severity of proteinuria. Glomerular CD169 ^+^ macrophages decreased in number after corticosteroid treatment ^71^.

B cells are central to LN pathogenesis since they are precursors for autoantibody-producing plasma cells, present antigens to T cells, and contribute to cytokine secretion. Vimentin expressed by infiltrating inflammatory cells has been reported to serve as an autoantigen that induced *in situ* B-cell selection during tubulo-interstitial inflammation in LN ^72^. Activated macrophages secrete vimentin in response to pro-inflammatory signaling ^73^ and may be a source of vimentin in the tubulo-interstitium. Alternatively, PTECs undergoing epithelial-to-mesenchymal transition (EMT) and apoptosis may also contribute to the vimentin repertoire in the kidney since vimentin is an intermediate filament synthesized by mesenchymal cells and is also expressed on the cell surface of apoptotic cells ^74, 75^. Post-translational modification of vimentin may increase its antigenicity and exacerbate autoimmunity ^76^. Elevated anti-vimentin antibody levels are observed in patients with LN, and the level of these antibodies correlated with tubulo-interstitial inflammation ^72^. Yet how these autoantibodies contribute to tubulo-interstitial injury remains to be investigated. Anti-vimentin antibodies have also been detected in a proportion of recipients with renal or cardiac allograft, and mycophenolate (MPA) treatment was associated with lower anti-vimentin antibody levels compared with azathioprine in cardiac allograft recipients ^77, 78^.

## Recent knowledge on renal fibrosis in lupus nephritis

Renal fibrosis is a common feature of chronic inflammatory disorders where wound-healing processes persist and become excessive, with prolonged production of growth factors, fibrogenic cytokines and proteolytic enzymes and their inhibitors, leading to increased synthesis and decreased degradation of the extracellular matrix. Both resident renal cells and immune cells contribute to kidney fibrosis.

### (a) Role of resident kidney cells

Appropriate regulation of inflammation is essential to prevent progressive kidney fibrosis in LN. Fibronectin is the predominant matrix protein present in glomerulosclerotic lesions and is one of the first matrix components to be deposited during tubulo-interstitial fibrosis ^79, 80^. Intra-glomerular fibronectin expression is increased in patients and mice with active LN ^15, 16^ and co-localizes with IgG deposition, suggesting an association between autoantibody deposition and matrix protein accumulation ^16^. We reported that anti-dsDNA antibodies can induce both soluble and fibrillar fibronectin synthesis in mesangial cells and PTECs through increased PKC and MAPK signaling and TGF-β1, MCP-1, IL-6, IL-8, and TNF-α secretion ^14, 16^. Our observation that pro-inflammatory mediators can also contribute to increased fibrogenesis highlights their multi-faceted functions during pathogenesis. The role of TGF-β1 as a key mediator of kidney fibrosis is well established ^81, 82^, but it is noteworthy that TGF-β1 also plays an important role in immune regulation and can suppress B-cell auto-reactivity during the early phase of disease but can exert pro-fibrotic actions when LN is established ^81, 83^. Fibrogenic cells are characterized by their ability to synthesize fibrillar collagen. We demonstrated that soluble fibronectin can induce TGF-β1 and collagen synthesis in PTECs, thereby amplifying the fibrogenic response of anti-dsDNA antibodies in the tubulo-interstitium ^14^. Increased fibronectin has been reported to induce EMT in lung alveolar epithelial cells ^84^. It is possible that anti-dsDNA antibody induction of fibronectin induces EMT in PTECs, although further studies are warranted to confirm this. In this regard, we have demonstrated that anti-dsDNA antibodies derived from patients with LN during active disease and remission can induce phenotypic changes in PTECs with the acquisition of an elongated, fibroblastic appearance ^10^. Myofibroblasts are the primary source of matrix proteins in renal fibrosis, and these cells may originate from PTECs undergoing EMT, interstitial fibroblasts, or pericytes ^85, 86^. There is emerging evidence that epigenetics may contribute to the progression of renal fibrosis, and miR-150 has been implicated in tubulo-interstitial fibrosis in patients with LN through its ability to repress suppressor of cytokine signaling 1 ^87^. In a murine model of chronic kidney disease, reduced fatty acid oxidation in renal tubular epithelial cells appeared to contribute to kidney fibrosis, possibly through mitochondrial dysfunction ^88^. The role of fatty acid oxidation in tubular epithelial cells and tubulo-interstitial fibrosis in LN is not known.

### (b) Role of infiltrating cells

Monocyte-derived macrophages play an important role in tissue fibrosis through direct effects on matrix remodeling or indirectly through the activation of myofibroblasts. Unlike pro-inflammatory M1 macrophages, M2 macrophages exert anti-inflammatory responses and contribute to reparative processes following tissue injury. In immune-mediated injury where the inciting factors persist, M2 macrophages drives fibrogenesis through increased synthesis of growth factors, polyamine and proline ^89^ and the generation of a provisional matrix that promotes recruitment and activation of fibroblasts. In animal studies, depletion of macrophages has been shown to reduce the severity of crescentic glomerulonephritis and tubulo-interstitial fibrosis ^90– 92^. Bone marrow-derived M2 macrophages can undergo myofibroblastic transition and contribute to collagen and α-smooth muscle actin expression in areas of kidney fibrosis in patients with IgA nephropathy or rapidly progressive glomerulonephritis ^93^. It is possible that M2 macrophages also contribute to kidney fibrosis through a similar mechanism in LN. Although α-smooth muscle actin is often associated with myofibroblasts, there is evidence that it is also expressed in the kidney of healthy subjects and neonatal pericytes and thus is not a reliable marker for myofibroblasts ^94^. Instead, fibrillar collagen has been suggested to be a more appropriate marker ^94^. Of note, renal myofibroblasts lacking α-smooth muscle actin expression are associated with increased cell proliferation and collagen production and have been reported to contribute to renal fibrosis ^95^.

## Current and emerging treatments for lupus nephritis and their effects on inflammatory and fibrotic processes

Preservation of nephrons is critical in ensuring long-term renal and patient survival ^1^. The current standard-of-care induction treatments for severe LN are corticosteroids combined with either cyclophosphamide (CYC) or MPA ^96– 99^. We have reported that MPA can exert a beneficial role on inflammatory and fibrotic processes induced by anti-dsDNA antibodies in human mesangial cells and PTECs and that this role is independent of its immunosuppressive actions ^14– 16, 39^. We have also demonstrated that MPA together with methylprednisolone (MP) was more effective than CYC and MP in preserving renal histology with reduced severity of renal fibrosis in New Zealand black and white first-generation (NZB/W F1) mice, possibly through the ability of MPA to decrease PKC-α activation and TGF-β1 expression ^15^. Long-term follow-up studies have reported a relatively high incidence of chronic kidney failure in patients with LN previously treated with CYC, especially in subjects with a greater propensity for renal fibrosis, such as African-Americans ^100– 102^. In a murine model of progressive renal interstitial fibrosis, CYC treatment alone was shown to induce interstitial fibrosis, which was associated with the depletion of macrophages, although the subtype was not determined ^103^. Whether exposure to CYC in susceptible individuals tips the balance in favor of fibrosis instead of repair is an intriguing possibility with significant clinical implications. In patients with neoplastic diseases, CYC treatment was associated with urinary bladder inflammation and fibrosis, and the severity of fibrosis was associated with the dose and duration of CYC treatment ^104^. It was unclear whether the bladder fibrosis was consequent solely to CYC-induced uroepithelial inflammation or was aggravated by a separate pro-fibrotic effect of CYC. In addition to MMF and CYC, azathioprine and calcineurin inhibitors are used in the treatment of lupus. Whether these pharmacologic agents, in addition to their immunosuppressive actions, can exert a direct effect on kidney inflammation has not been investigated. In contrast, the chronic nephrotoxicity of calcineurin inhibitors has been investigated in organ transplant recipients and is characterized by renal parenchymal fibrosis, vascular hyalinization and prominent induction of TGF-β ^105^. Angiotensin-converting enzyme (ACE) inhibitors and angiotensin II receptor blockers have been shown to reduce proteinuria and the rate of renal function deterioration in patients with chronic glomerular diseases such as diabetic nephropathy or IgA nephropathy. In patients with quiescent LN and persistent proteinuria, treatment with ACE inhibitors or angiotensin II receptor blockers resulted in sustained improvements in proteinuria and serum albumin level ^106^. Treatment of lupus-prone mice with ACE inhibitors delayed the onset of proteinuria, reduced disease progression and chronic kidney lesions, and was associated with decreased intra-glomerular expression of TGF-β1 and TGF-β2 and splenic production of type 2 cytokines such as IL-4 and IL-10 ^107^. It is conceivable that treatment of lupus patients who have chronic renal damage with blockade of the renin-angiotensin pathway should exert a similar beneficial effect on kidney fibrosis.

Although high-dose corticosteroids remain a cornerstone in the treatment of severe active LN, the use of pulse corticosteroids is highly variable between clinicians ^96– 99^. Intravenous pulse corticosteroid treatment has been reported to be more effective than oral corticosteroids in suppressing circulating and intra-renal expression of pro-inflammatory cytokines in autoimmune conditions, including LN ^108– 110^. Although this should theoretically be accompanied by reduction of renal inflammation, the impact of pulse corticosteroids on renal inflammation and fibrosis remains to be examined.

Biological therapies target key molecules in pathogenic pathways, based on knowledge of the immunopathogenic mechanisms in LN. Biologics tested or being developed in SLE or LN (or both) inhibit B-cell proliferation and activation (for example, anti-BAFF), target B-cell subpopulations (for example, anti-CD20), reduce co-stimulatory signal in T lymphocyte activation (for example, cytotoxic T-lymphocyte associated protein 4 (CTLA-4) Ig), or antagonize the effect of key cytokines (for example, IFN-α) ^111– 115^. Treatment of lupus-prone mice with anti-BAFF or CTLA-4 Ig was shown to ameliorate glomerular inflammation and tubular damage and decrease intra-renal inflammatory cytokine expression ^116, 117^. Anti-IL-6 monoclonal antibodies were shown to ameliorate nephritis in murine LN models ^118, 119^; yet in a controlled trial, the anti-IL-6 antibody sirukumab given for 24 weeks did not reduce proteinuria that persisted despite standard induction immunosuppressive treatment for LN, and the treatment was associated with excessive serious infections ^120^. TWEAK (TNF-like weak inducer of apoptosis) is a member of the TNF family of cytokines, and the Fn14 gene codes for the TWEAK receptor. Monocytes, dendritic cells, and natural killer cells are the major sources of TWEAK. Although transient activation of the TWEAK/Fn14 pathway is involved in tissue repair after injury, excessive or persistent activation of the pathway is implicated in autoimmune diseases, including lupus, and activation of the pathway in diseased organs has been reported to drive local inflammation leading to fibrosis ^121^. Anti-TWEAK antibody therapy has yielded promising results in animal models of autoimmune diseases ^122, 123^. A phase II randomized placebo-controlled clinical trial that explored the efficacy, tolerability, and safety of anti-TWEAK antibody as an add-on therapy in patients with class III/IV LN did not demonstrate sufficient efficacy, and the drug development program was terminated (ATLAS study, NCT01499355).

Apart from positive trial outcomes with belimumab and anifrolumab in lupus patients without severe nephritis ^111, 112, 124^, the other clinical trials on biologics to date either failed to achieve the efficacy study endpoints or have been aborted because of perceived unfavorable balance in effect size versus adverse events ^114, 115, 125^. The data nevertheless suggest that a subset of patients could derive clinically observable benefit when these agents were added to conventional immunosuppressive therapies for active LN ^113– 115^. Factors contributing to the apparent discrepancy between biological effect and clinical outcomes include efficacy of background therapy, definition of clinical study outcomes ^126^, and patient heterogeneity, the last of which was evidenced by the greater impact of rituximab treatment in African-Americans compared with Caucasians ^114^. The impact of these agents on renal parenchymal inflammation or fibrosis remains unknown as this is rarely an outcome parameter and the data have not been systematically examined.

## Conclusions

Inflammation and fibrosis are key processes in LN and involve the interplay of immune cells of the innate and adaptive immune system and resident renal cells. The identification of molecules or pathways that contribute to the development of LN has flourished over the past decade and is ever-expanding. Identifying the roles of inflammatory mediators and the molecular mechanism that regulate inflammatory responses is made more challenging by their multi-faceted roles, not only at the onset of pathogenesis but also during the effector phase where they facilitate kidney injury. Defining how these molecules contribute to disease pathogenesis is crucial before more focused therapeutic strategies can be devised. Despite a sound mechanistic rationale and encouraging animal data, the clinical results with biologics have been disappointing to date, yet the failure to demonstrate clinical utility could reflect deficiencies in protocol design rather than a lack of biological effect. There are a number of murine models of LN, and choosing an optimal model is imperative to identify key checkpoints of kidney injury and in the evaluation of potential therapeutic interventions. Table 1 summarizes the phenotypic differences between different murine models of LN. Current therapies primarily target immunological and inflammatory pathways, yet disease mechanisms that lead to myofibroblast conversion and fibrotic processes should not be overlooked. Lastly, given that a multitude of effector mechanisms are activated in patients with LN, biological profiling of patients with a proteomics or genomics approach (or both) may facilitate better selection of treatment regimens.

**Table 1.** Phenotypic differences between different murine models of lupus nephritis.

	Strain/ model	Clinical manifestations	Anti-dsDNA antibody production	Renal histological changes	References
Spontaneous lupus nephritis models	NZB/W F1	• Disease is more severe and occurs earlier in female mice. • 50% mortality at 8 months in female mice and at 15 months in male mice • Proteinuria appears at 5 months. • Death due to immune-mediated GN	High titres of IgG anti-dsDNA antibodies detected by 4–5 months of age	• Deposition of IgG and C3 in glomerular and tubular basement membranes • Proliferative changes in mesangial and endothelial cells • Thickening of glomerular basement membrane • Increased glomerular and tubulo-interstitial expression of PKC-α, PKC-βI, PKC-βII, TGF-β1, collagen, and fibronectin • Glomerulosclerosis • Mononuclear cell infiltration into interstitium • Increased cytokine and chemokine expression • Increased tubulo-interstitial activation of MAPK signaling pathway • Tubulo-interstitial fibrosis • Tubular atrophy	15, 16, 39, 127– 129
	NZM2328	• Disease is more severe in female mice.	High titres of IgG anti- dsDNA antibodies	• Glomerulosclerosis • Tubular atrophy • Congenic NZM.C57Lc1 mice develop severe chronic GN without antinuclear antibody production	127, 130
	NZM2410	• Disease manifestations similar to NZB/W F1 mice • Disease severity similar in female and male mice	High titres of IgG anti- dsDNA antibodies	• Early onset of glomerulosclerosis compared with NZB/W F1 and MRL/lpr mice • Deposition of IgG and C3 in glomerular and tubular basement membranes • Proliferative changes in mesangial and endothelial cells • Thickening of glomerular basement membrane • Glomerulosclerosis • Tubulo-interstitial fibrosis • Tubular atrophy	127
	MRL/lpr	• Marked lymphadenopathy and splenomegaly • 50% mortality at 6 months in female and male mice • Proteinuria appears at 1–3 months in female mice. • Death due to immune-mediated GN	High titres of IgG anti-dsDNA antibodies detected at 6–8 weeks of age	• Deposition of IgG and C3 in glomerular and tubular basement membranes • Subacute proliferative changes in mesangial and endothelial cells • Thickening of glomerular basement membrane • Crescent formation • Mononuclear cell infiltration into interstitium • Increased cytokine and chemokine expression • Increased collagen accumulation in glomerular and tubular basement membranes	127– 129, 131
	BXSB	• Disease predominance in male mice • Late onset of lupus in female mice • 50% mortality at 5 months in male mice and at 15 months in female mice • Proteinuria appears by 3 months. • Death due to immune-mediated GN	High titres of IgG anti- dsDNA antibodies	• Deposition of IgG and C3 in glomerular and tubular basement membranes • Acute to subacute GN • Exudation of neutrophils into the glomerulus • Subacute proliferative changes in mesangial and endothelial cells • Thickening of glomerular basement membrane • Development of proliferative nephritis	127– 129, 132
Induction of lupus nephritis in non-autoimmune mice	Pristane	• Female mice more susceptible to disease • Nephritis occurs 6 months after pristane-injection, although development of nephritis is variable. • Moderate proteinuria	IgM and not IgG anti -ssDNA antibodies detected within 1–4 weeks of pristane injection	• Development of renal disease more severe in BALB/c mice than in C57BL/6 mice • Focal to global proliferative lesions • Effacement of capillaries • Glomerular basement membrane thickening • Deposition of IgG, IgM, and C3 in mesangial and subendothelial area • Immune complexes in these mice are not attributed to IgG anti-dsDNA antibodies.	133– 136
	Chronic graft- versus-host disease	• 50% mortality between 9 and 16 weeks post-induction • Moderate proteinuria observed 2 weeks post-induction with slow increase up to 6 weeks	IgG anti-dsDNA antibodies detected	• Immune complexes detected in glomeruli 4–5 weeks post-induction • C57BL/6 mice develop mild nephritis with antibody deposition in mesangial cell (class II). • BALB/c and SJL mice develop proliferative lupus nephritis with IgG deposition present along the capillary loops. • Membranous nephritis • Electron-dense deposits in the mesangium, subendothelial, and subepithelial regions • Induction of pro-inflammatory cytokines through upregulation of TWEAK	127, 135, 137, 138

C3, complement 3; dsDNA, double-stranded DNA; GN, glomerulonephritis; Ig, immunoglobulin; MAPK, mitogen-activated protein kinase; NZB/W F1, New Zealand black and white first generation; PKC, protein kinase C; ssDNA, single-stranded DNA; TGF-β1, transforming growth factor-beta 1; TWEAK, tumor necrosis factor-like weak inducer of apoptosis.

## Abbreviations

ACE, angiotensin-converting enzyme; BAFF, B cell-activating factor; CTLA-4, cytotoxic T-lymphocyte associated protein 4; CYC, cyclophosphamide; dsDNA, double-stranded DNA; EMT, epithelial-to-mesenchymal transition; FcR, Fc receptor; GBM, glomerular basement membrane; IFN, interferon; Ig, immunoglobulin; IL, interleukin; LN, lupus nephritis; MAPK, mitogen-activated protein kinase; MCP-1, monocyte chemoattractant protein-1; miR, microRNA; MP, methylprednisolone; MPA, mycophenolate; NF-κB, nuclear factor kappa B; PBMC, peripheral blood mononuclear cell; PI3K, phosphoinositide 3-kinase; PKC, protein kinase C; PTEC, proximal tubular epithelial cell; SLE, systemic lupus erythematosus; TGF-β1, transforming growth factor-beta 1; TLR, Toll-like receptor; TNF-α, tumor necrosis factor-alpha; TWEAK, TNF-like weak inducer of apoptosis.
